# Whole-Transcriptome Profiling on Small FFPE Samples: Which Sequencing Kit Should Be Used?

**DOI:** 10.3390/cimb44050148

**Published:** 2022-05-13

**Authors:** Marc Hilmi, Lucile Armenoult, Mira Ayadi, Rémy Nicolle

**Affiliations:** 1 Molecular Oncology, PSL Research University, CNRS, UMR 144, Institut Curie, 75005 Paris, France; marc.hilmi@curie.fr; 2 Programme Cartes D’Identité des Tumeurs (CIT), Ligue Nationale Contre le Cancer, 75013 Paris, France; lucile.armenoult@live.fr (L.A.); mira.ayadi@gmail.com (M.A.); 3 Centre de Recherche sur l’Inflammation (CRI), Université de Paris Cité, INSERM, U1149, CNRS, ERL 8252, 75018 Paris, France

**Keywords:** RNA sequencing, formalin-fixed paraffin-embedded samples, library preparation

## Abstract

RNA sequencing (RNA-Seq) appears as a great tool with huge clinical potential, particularly in oncology. However, sufficient sample size is often a limiting factor and the vast majority of samples from patients with cancer are formalin-fixed paraffin-embedded (FFPE). To date, several sequencing kits are proposed for FFPE samples yet no comparison on low quantities were performed. To select the most reliable, cost-effective, and relevant RNA-Seq approach, we applied five FFPE-compatible kits (based on 3′ capture, exome-capture and ribodepletion approaches) using 8 ng to 400 ng of FFPE-derived RNA and compared them to Nanostring on FFPE samples and to a reference PolyA (Truseq) approach on flash-frozen samples of the same tumors. We compared gene expression correlations and reproducibility. The Smarter Pico V3 ribodepletion approach appeared systematically the most comparable to Nanostring and Truseq (*p* < 0.001) and was a highly reproducible technique. In comparison with exome-capture and 3′ kits, the Smarter appeared more comparable to Truseq (*p* < 0.001). Overall, our results suggest that the Smarter is the most robust RNA-Seq technique to study small FFPE samples and 3′ Lexogen presents an interesting quality–price ratio for samples with less limiting quantities.

## 1. Introduction

High throughput sequencing is a keystone of precision and predictive medicine with most approach relying on analysis of DNA alterations [1,2]. RNA sequencing (RNA-Seq) is revolutionizing the study of the transcriptome by measuring gene expression across the entire genome in a highly sensitive and accurate way. In addition to gene expression, RNA-Seq may allow the detection of transcript isoforms, gene fusions, and single nucleotide variants. Determination of these parameters make RNA-Seq a great tool with huge clinical potential, in particular in oncology [2,3]. Another application of transcriptome profiling is based on RNA signatures, providing a multiparametric score based on several gene expressions. An increasing body of evidence shows the clinical interest of whole-transcriptome profiling for tumor phenotyping [4] and predictive response to treatment [5,6].

Standard RNA sequencing approaches require large amounts of high-quality RNA. The best quality RNAs are often obtained when samples are flash-frozen, which is not compatible with routine clinical practice in most centers. Clinical applicability of RNA-Seq is thus conditioned on its applicability to formalin-fixed paraffin-embedded (FFPE) samples. Moreover, sample quantity is highly variable. For biopsies performed on some organs (e.g., lung or pancreatic cancer), the tissue obtained from fine-needle biopsies can be highly limiting in quantity. This commonly used approach leads to only thousands of cells in samples that are often only available in FFPE, therefore with low RNA quality (RNA integrity number < 3) [7]. This requires RNA sequencing kits that are quantitative and reproducible with low quantities of low-quality RNA to be compatible with clinical routine.

To date, several sequencing kits are proposed to be compatible with FFPE samples with little data comparing them, especially in a low quantity setting. It has been previously reported that transcriptome analyses depend on the sequencing kits used [8,9,10]. On one hand, the most reliable technology for quantifying RNA is Nanostring as it directly quantifies RNA whereas other technologies quantify cDNA after reverse transcription. However, Nanostring quantifies a limited number of genes restricting the application and the possibility to improve signatures (e.g., molecular subtypes, theranostic, and immune-related signatures). On the other hand, PolyA enrichment is the reference efficient technology for RNA sequencing but is not compatible with FFPE samples.

In order to select the most reliable, cost-effective, and relevant RNA-Seq approaches, we applied several sequencing kits marketed as FFPE-compatible and compared their expression profiles to Nanostring quantification of a set of selected genes and to poly-A enriched RNAseq profiles of the flash-frozen samples of the same tumor.

## 2. Materials and Methods

### 2.1. Design of the Study

The RNA was extracted from 20 breast cancer tumors for which a flash-frozen and FFPE sample was available. Six different RNA-sequencing kits were used. Illumina’s TruSeq polyA enrichment kit (RNA Library Prep Kit v2, referred to as TruSeq) was applied to RNA derived from the frozen samples and used as a reference. Illumina’s TruSeq RNA exome (referred to as RNAaccess) was applied to 12 of the 20 FFPE samples. Lexogen’s QuantSeq 3′ approach (hereby referred to as Lexogen) was applied to FFPE samples using a range of input quantities, 50 ng, 150 ng, and 400 ng. Three ribodepletion approaches were applied each to FFPE-derived RNA: SMARTer Stranded Total RNA-Seq Kit v3-Pico, Ovation SoLo and SEQuoia Complete Stranded RNA (respectively referred to as Smarter, Solovation, and Sequoia). For the three ribodepletion kits, four samples were replicated. Finally, a Nanostring nCounter panel of the molecular subtypes of breast cancer (91 genes) was applied to FFPE-derived RNA.

### 2.2. Samples

In total, 20 breast tumors, for which both frozen and a formalin-fixed paraffine embedded tissue sample, were available were obtained from the Institut regional du cancer Montpellier. RNA was extracted using trizol and Guanidine Isothiocyanate and isolated using RNeasy Mini kit (Qiagen) and the ALLPrep FFPE tissue kit (Qiagen) for frozen and FFPE samples, respectively, following the manufacturer’s instruction. The quality of FFPE-derived RNA was measured by the proportion of fragments above 200b (DV200) and ranged from 13% to 69% with an average of 42%. The FFPE-derived RNA of all 20 samples were used on 4 FFPE-compatible RNAseq library preparation: Lexogene’s QuantSeq FWD 3′ kit, BIO-RAD’s SEQuoia complete stranded RNA library kit, Takara’s SMARTer Stranded total RNA-seq kit v3-pico (Smarter), and TECAN’s Ovation SoLo (Solovation) RNA-Seq library preparation kit.

The following total quantities of FFPE-derived RNA were used (Table 1): 400 ng for RNAAccess, 50 ng, 150 ng, or 400 ng for Lexogene, 26 ng for Sequoia, 5 ng for Solovation and 8 ng for SMARTER. For the Sequoia, Solovation and SMARTER, 4 samples were also replicated using 2 ng of FFPE-derived RNA. The 400 ng of frozen-derived RNA from 20 samples was used for the TruSeq library preparation. All kits were applied following vendor’s instructions. Illumina short-read sequencing was performed aiming for 20 million paired-end reads for each kit, except Lexogene’s 3′ kit which was run aiming for 10 million single-end reads.

A nCounter Nanostring breast cancer gene panel was applied to 150 ng of FFPE-derived RNA.

### 2.3. RNA Sequence Processing

Sequencing reads were mapped to the human genome GRCh38 (Ensembl v101) and Ensembl’s reference transcriptome (v101) using a single-pass of STAR with the following arguments changed from their default: seedSearchSartLmax 12, outFilterType BySJout and alignEndsType Local. FeatureCount was used to count RNAseq reads per any overlapping exon (-O argument) and then summarized per gene (as meta-features) using appropriate manufacturer defined stranding. Raw gene-level RNA counts were then normalized using the upper-quartile normalization and log2 + 1 transformed.

### 2.4. Statistical Tests

Gene-wise and sample-wise correlations of gene expressions was performed using a Spearman correlation test. Comparison of genes expressions and sample coefficients correlation between RNAAccess and other sequencing kits (Lexogen, Solovation, Sequoia, Smarter) was performed using a Mann–Whitney test. For each test, statistical significance was set at a two-sided *p* value of <0.05.

## 3. Results

### 3.1. Number of Expressed Genes

**Figure 1:**
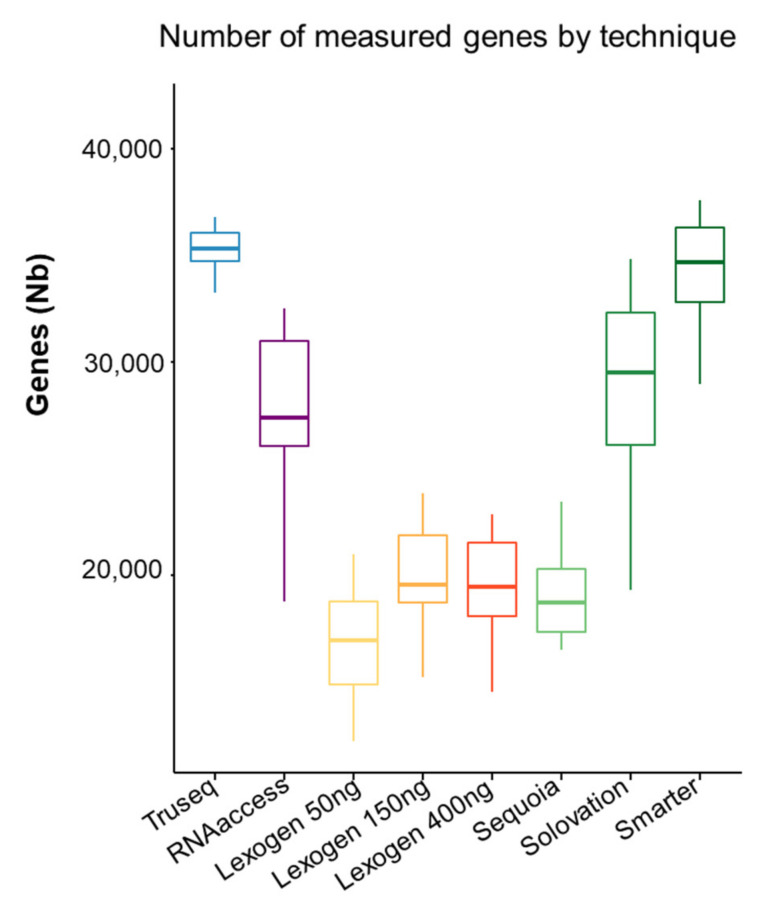
Number of expressed genes according to RNA sequencing kits.

The number of genes with at least one count per sample by each technique is shown in Figure 1.

Truseq and Smarter quantified the largest number of genes (mean of 35′032 and 34′372, respectively) whereas Lexogen using 50 ng of RNA and Sequoia identified about 2 times less (mean of 16′764 and 18′864, respectively).

### 3.2. Comparison with Nanostring

**Figure 2:**
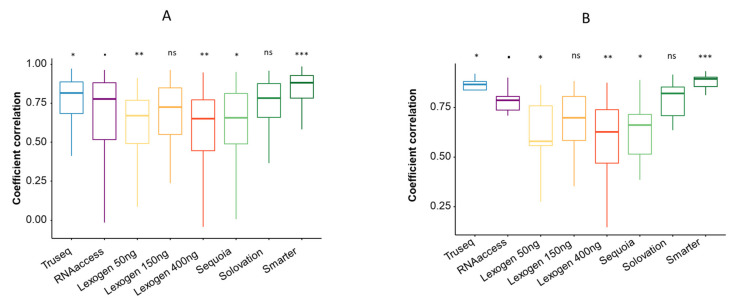
Comparison of sequencing kits with Nanostring. (**A**) Gene-wise correlation of gene expressions between Nanostring and other sequencing kits. The distribution of the correlation coefficients for each technique were compared using a Mann–Whitney test and using RNAAccess as a comparison. The lower and upper hinges correspond to the first and third quartiles. The upper whisker extends from the hinge to the largest value no further than 1.5 * interquartile range from the hinge. The lower whisker extends from the hinge to the smallest value at most 1.5 * interquartile range of the hinge. ^.^: reference; *: *p*-value between 0.01 and 0.05. **: *p*-value between 0.001 and 0.01; ***: *p*-value < 0.001. (**B**) Sample-wise correlation between Nanostring and other sequencing kits. The distribution of the correlation coefficients for each technique were compared using a Mann–Whitney test and using RNAAccess as a comparison. The lower and upper hinges correspond to the first and third quartiles. The upper whisker extends from the hinge to the largest value no further than 1.5 * interquartile range from the hinge. The lower whisker extends from the hinge to the smallest value at most 1.5 * interquartile range of the hinge. ^.^: reference; *: *p*-value between 0.01 and 0.05. **: *p*-value between 0.001 and 0.01; ***: *p*-value < 0.001.

In order to evaluate the accuracy of gene expression quantification, the transcriptomic profiles were first correlated to those obtained by Nanostring using a gene-wise correlation (Figure 2A). In this setting, we observed that Smarter and Truseq were the most correlated with Nanostring (mean coefficient correlation 0.816 and 0.759, respectively). By comparing coefficient correlations of each technique using a Mann–Whitney test and using RNAAccess as a comparison, we found a significantly lesser correlation by Lexogen 50 ng (*p* = 0.006), Lexogen 400 ng (*p* = 0.004), Sequoia (*p* = 0.02), and a significantly higher correlation with Nanostring using Truseq (*p* = 0.05) and Smarter (*p* < 0.0001).

Secondly, we correlated each profile with its Nanostring reference for each technique (sample-wise correlation), focusing only on the genes composing the panel (Figure 2B). By comparing sample correlation using a Mann–Whitney test among each technique and using RNAAccess as reference, we found the same findings than the gene expression correlation.

### 3.3. Comparison with Truseq

In order to perform a broader analysis, we used the frozen-based Truseq profiles as reference. In order to take into account the potential difficulty to quantify lowly expressed genes in FFPE, genes were split in three groups (tercile) based on their median expression levels in Truseq. The upper and lower terciles were 3.7 and 9.0 log2 counts, respectively.

**Figure 3:**
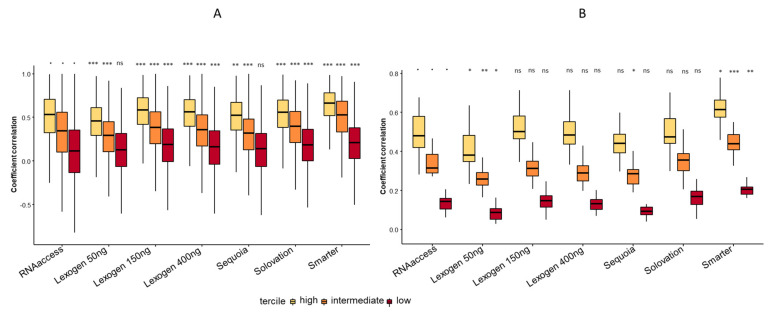
Comparison of sequencing kits with Truseq. (**A**) Gene-wise correlation of gene expressions between Truseq and other sequencing kits. Coefficient correlations of each technique in every tercile were compared using a Mann–Whitney test and using RNAAccess as a comparison. The lower and upper hinges correspond to the first and third quartiles. The upper whisker extends from the hinge to the largest value no further than 1.5 * interquartile range from the hinge. The lower whisker extends from the hinge to the smallest value at most 1.5 * interquartile range of the hinge. ^.^: reference; *: *p*-value between 0.01 and 0.05. **: *p*-value between 0.001 and 0.01; ***: *p*-value < 0.001. (**B**) Sample-wise correlation of samples between Truseq and other sequencing kits. Coefficient correlations of each technique in every tercile were compared using a Mann–Whitney test and using RNAAccess as a comparison. The lower and upper hinges correspond to the first and third quartiles. The upper whisker extends from the hinge to the largest value no further than 1.5 * interquartile range from the hinge. The lower whisker extends from the hinge to the smallest value at most 1.5 * interquartile range of the hinge. ^.^: reference; *: *p*-value between 0.01 and 0.05. **: *p*-value between 0.001 and 0.01; ***: *p*-value < 0.001.

First, we correlated gene levels expression between Truseq and each technique (gene-wise correlation, Figure 3A). The highest tercile of gene correlated the most compared to other terciles and Smarter was the most quantitative technique. By comparing coefficient of correlations using a Mann–Whitney test among each technique in the lowest tercile using RNAAccess as reference, we found a higher correlation with Lexogen 150 ng, Lexogen 400 ng, Solovation and Smarter (*p* < 0.0001). In the intermediate and highest terciles, the correlation with Truseq was higher with Lexogen 150 ng, Lexogen 400 ng, Solovation and Smarter (*p* < 0.0001), and less robust with Lexogen 50 ng and Sequoia (*p* < 0.0001).

We then performed sample-wise correlation between Truseq and each technique (Figure 3B). We analyzed each tercile by comparing sample correlation using a Mann–Whitney test among each technique using RNAAccess as reference. In all terciles, we found that Lexogen 50 ng was significantly less correlated (*p* < 0.05) and Smarter showed higher correlation with Truseq (*p* < 0.05).

Finally, in all terciles and by comparing gene or samples, Smarter on small quantities of FFPE-derived RNA had the best correlation with Truseq applied to standard quantities of Frozen-derived RNA.

### 3.4. Reproducibility

**Figure 4:**
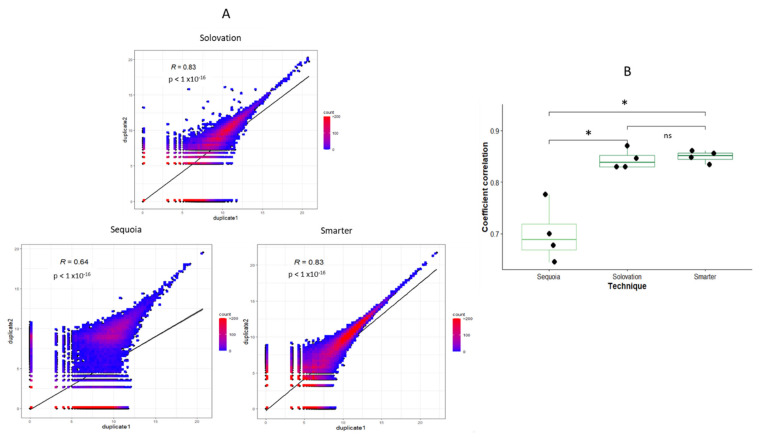
Reproducibility among Sequoia, Solovation, and Smarter sequencing kits. (**A**) Correlation with Spearman test of gene expression between replicates of the same kit. (**B**) Correlation between 4 replicates for Sequoia, Solovation, and Smarter. Coefficient correlations of each technique were compared using a Mann–Whitney test. The lower and upper hinges correspond to the first and third quartiles. The upper whisker extends from the hinge to the largest value no further than 1.5 * interquartile range from the hinge. The lower whisker extends from the hinge to the smallest value at most 1.5 * interquartile range of the hinge.

The kit reproducibility was assessed for Sequoia, Solovation, and Smarter by correlating gene expression profiles with duplicates of lower input quantity (2 ng) (Figure 4A). Correlation using a Mann–Whitney test was significantly higher between Smarter and Solovation replicates than Sequoia (*p* = 0.029). Distribution of correlation between each replicate for each sequencing kit is presented in Figure 4B.

## 4. Discussion

This study aims to compare recent RNA-sequencing library kits suggested by vendors as compatible with FFPE, and for ribodepletion kits, with small quantities of FFPE-derived RNA. A 3′ capture for standard RNA quantities and several ribodepletions techniques for quantities below 10 ng of total RNA were used. Although ribodepletion techniques were compatible with low quantities out of the box, we tested the lower limits of 3′ captures to test their applicability to small samples. The previous FFPE RNAseq approach RNAaccess was also applied as a historical reference. By analyzing gene-wise and sample-wise correlations of gene expression against a reference frozen polyA RNAseq or an FFPE-derived Nanostring panel, Smarter’s Pico V3 appeared systematically the most reliable sequencing kit and one of the most reproducible technique. These results are particularly relevant since Smarter performed well on small amounts of FFPE-derived total RNA. Previous studies assessing performance of different library prep also showed that Smarter’s kit was the most comparable with Truseq among other kits [9,11,12,13]. The 3′ Lexogen using 150 and 400 ng appears a cost-efficient whole transcriptome technique with a library preparation approximately half the price and requiring approximately three times less sequences compared to other techniques.

The main limit of our study is that samples are obtained from breast cancers only. RNA-seq performance may also be dependent on the type of tissue and we did not have other tissue to validate our results. However, we can assume that low-quality RNA related to specific tissues (e.g., bone, pancreas) affect similarly all sequencing kits.

## 5. Conclusions

Overall, the Pico V3 Smarter approach should be preferred to study the transcriptome of FFPE samples whereas Lexogen presents an interesting quality price ratio for large quantities of RNA.

## Figures and Tables

**Table 1:** Summary of the features of each sequencing kit.

Sequencing Kit	Methodology	Number of Samples	Amount of RNA Used (ng)
Nanostring	nCounter	20	150
Trueseq	PolyA enrichment	20	400
RNAAccess	Exome capture	12	400
Lexogen 50 ng	3′ polyA capture	20	50
Lexogen 150 ng	3′ polyA capture	20	150
Lexogen 400 ng	3′ polyA capture	20	400
Sequoia	Ribodepletion	20 (+4 rep.)	26 (rep. 2 ng)
Solovation	Ribodepletion	20 (+4 rep.)	5 (rep. 2 ng)
Smarter	Ribodepletion	20 (+4 rep.)	8 (rep. 2 ng)

rep.: replicates.

## Data Availability

Data available on demand.

## References

[1] Moorcraft S.Y., Gonzalez D., Walker B.A. (2015). Understanding next generation sequencing in oncology: A guide for oncologists. Crit. Rev. Oncol..

[2] Malone E.R., Oliva M., Sabatini P.J.B., Stockley T.L., Siu L.L. (2020). Molecular profiling for precision cancer therapies. Genome Med..

[3] Bertucci F., Ng C.K.Y., Patsouris A., Droin N., Piscuoglio S., Carbuccia N., Soria J.C., Dien A.T., Adnani Y., Kamal M. (2019). Genomic characterization of metastatic breast cancers. Nature.

[4] Rodon J., Soria J.C., Berger R., Miller W.H., Rubin E., Kugel A., Tsimberidou A., Saintigny P., Ackerstein A., Braña I. (2019). Genomic and transcriptomic profiling expands precision cancer medicine: The WINTHER trial. Nat. Med..

[5] Nicolle R., Gayet O., Duconseil P., Vanbrugghe C., Roques J., Bigonnet M., Blum Y., Elarouci N., Armenoult L., Ayadi M. (2021). A transcriptomic signature to predict adjuvant gemcitabine sensitivity in pancreatic adenocarcinoma. Ann. Oncol..

[6] Sparano J.A., Gray R. (2019). TAILORx: Questions Answered, Lessons Learned, and Remaining Knowledge Gaps. J. Clin. Oncol..

[7] Von Ahlfen S., Missel A., Bendrat K., Schlumpberger M. (2007). Determinants of RNA quality from FFPE samples. PLoS ONE.

[8] Park Y.S., Kim S., Park D.G., Kim D.H., Yoon K.W., Shin W., Han K. (2019). Comparison of library construction kits for mRNA sequencing in the Illumina platform. Genes Genom..

[9] Song Y., Milon B., Ott S., Zhao X., Sadzewicz L., Shetty A., Boger E.T., Tallon L.J., Morell R.J., Mahurkar A. (2018). A comparative analysis of library prep approaches for sequencing low input translatome samples. BMC Genom..

[10] Wong R.K.Y., MacMahon M., Woodside J.V., Simpson D.A. (2019). A comparison of RNA extraction and sequencing protocols for detection of small RNAs in plasma. BMC Genom..

[11] Sarantopoulou D., Tang S.Y., Ricciotti E., Lahens N.F., Lekkas D., Schug J., Guo X.S., Paschos G.K., FitzGerald G.A., Pack A.I. (2019). Comparative evaluation of RNA-Seq library preparation methods for strand-specificity and low input. Sci. Rep..

[12] Lin X., Qiu L., Song X., Hou J., Chen W., Zhao J. (2019). A comparative analysis of RNA sequencing methods with ribosome RNA depletion for degraded and low-input total RNA from formalin-fixed and paraffin-embedded samples. BMC Genom..

[13] Alberti A., Belser C., Engelen S., Bertrand L., Orvain C., Brinas L., Cruaud C., Giraut L., Da Silva C., Firmo C. (2014). Comparison of library preparation methods reveals their impact on interpretation of metatranscriptomic data. BMC Genom..

